# Transcriptome analyses of Acer Truncatum Bunge seeds to delineate the genes involved in fatty acid metabolism

**DOI:** 10.1186/s12864-024-10481-1

**Published:** 2024-06-17

**Authors:** Liping Yan, Hongcheng Fang, Yan Liang, Yinhua Wang, Fei Ren, Xiaoman Xie, Dejun Wu

**Affiliations:** 1 Shandong Provincial Key Laboratory of Forest Tree Genetic Improvement, Shandong Provincial Academy of Forestry, Jinan, Shandong China; 2 College of Forestry, Shandong Agricultural University, Tai’an, Shandong China; 3 Shandong Provincial Forest and Grass Germplasm Resources Center, Jinan, Shandong China

**Keywords:** *Acer Truncatum* Bunge, Fatty acid, Nervonic acid, Transcriptome

## Abstract

**Background:**

*Acer truncatum* Bunge is an economic, ecological, oil, and medicinal tree, and its kernel oil is rich in nervonic acid. It is crucial to explore the transcriptional expression patterns of genes affecting fatty acid synthesis to improve the quality of *Acer truncatum* oil.

**Results:**

This study used the seeds from high fatty acid strain YQC and those from low fatty acid strain Y38 as the test materials. Specifically, we performed a comparative transcriptome analysis of Y38 seeds and YQC to identify differentially expressed genes (DEGs) at two time points (seeds 30 days after the blooming period and 90 days after the blooming period). Compared with YQC_1 (YQC seeds at 30 days after the blooming period), a total of 3,618 DEGs were identified, including 2,333 up-regulated and 1,285 downregulated DEGs in Y38_1 (Y38 seeds at 30 days after blooming period). In the Y38_2 (Y38 seeds at 90 days after the blooming period) versus YQC_2 (YQC seeds at 90 days after the blooming period) comparison group, 9,340 genes were differentially expressed, including 5,422 up-regulated and 3,918 down-regulated genes. The number of DEGs in Y38 compared to YQC was significantly higher in the late stages of seed development. Gene functional enrichment analyses showed that the DEGs were mainly involved in the fatty acid biosynthesis pathway. And two fatty acid synthesis-related genes and seven nervonic acid synthesis-related genes were validated by qRT-PCR.

**Conclusions:**

This study provides a basis for further research on biosynthesizing fatty acids and nervonic acidnervonic acids in *A. truncatum* seeds.

**Supplementary Information:**

The online version contains supplementary material available at 10.1186/s12864-024-10481-1.

## Background

*Acer truncatum* Bunge is a crucial woody oil tree species in China, with various functions such as edible, medicinal, ornamental, chemical, and timber use [1] (Wei et al., 2018). The National Health Commission of the People’s Republic of China (PRC) (No. 9 Announcement issued in 2011) issued a notice approving *A*. *truncatum* seed oil as a new food raw material (http://www.nhc.gov.cn/). The oil content of *A. truncatum* seeds is 48%, of which 92% are unsaturated fatty acids, 53% are linoleic acid and linolenic acid, and 5.52% are nervonic acid (C24:1^Δ15^) [2–4]. Notably, nervonic acid is a very long-chain fatty acid (VLCFA) mainly present in brain tissues and nerves [5]. VLCFAs can reduce blood cholesterol and triglyceride levels, reducing cardiovascular disease risk [6]. Previous research has shown that the nervonic acid content in plasma and erythrocyte membranes was associated with neurological diseases [7].

The biosynthesis of VLCFAs is divided into two stages: de novo synthesis of fatty acids occurring in the plastids and fatty acid elongation occurring in the endoplasmic reticulum [8]. The de novo synthesis of fatty acids is mainly catalyzed by enzymes of the fatty acid synthase complex (β-ketoacyl-ACP synthase, KAS; β-ketoacyl-ACP reductase, KAR; β-hydroxyacyl-ACP dehydratase, DH; β-enoyl-ACP reductase, ENR), which undergoes four steps of condensation, reduction, dehydration, and reduction to form a cycle, and increased two carbon units to the carbon chain of fatty acids each cycle [9–11]. Free fatty acids are transported to the endoplasmic reticulum and enter the fatty acid elongation. Fatty acid elongation is mainly catalyzed by fatty acid elongase, which uses malonyl CoA as a 2 C donor and undergoes four steps to add two carbon units at the end of the fatty acid carbon chain. The fatty acid elongase complex (β-ketoacyl-CoA synthase, KCS; β-ketoacyl-CoA reductase, KCR; β-hydroxyacyl-CoA dehydratase, HCD; trans-2,3-enoyl-Co A reductase, ECR), sequentially participate in the carbon chain elongation reaction of fatty acids, ultimately generating VLCFAs [12, 13].

With the development of sequencing technology and bioinformatics analysis methods, candidate genes and pathways involved in the synthesis of fatty acids in different crops have been determined using transcriptome data. During the development of sesame (*Sesamum indicum* L.) seeds, the FAD2, LOC10515945, LOC105161564, and LOC105162196 genes were identified to regulate the accumulation of unsaturated fatty acid biosynthesis by the regulatory co-expression network [14]. In oil palm (*Elaeis guineensis* Jacq.), the transcriptome data revealed the expression profiles of genes in the fatty acid (FA) and triacylglycerol (TAG) biosynthesis processes in interspecific hybrids and identified the genes encoding key enzymes involved in the FA and TAG synthesis pathways [15]. Wang et al. analyzed the transcriptome of *A. truncatum* and categorized the enzymes (KCS, KCR, HCD, and ECR) involved in the biosynthesis of VLCFAs using high-throughput Illumina sequencing technology [16]. This study conducted the comparative transcriptome analysis of Y38 and YQC seeds to identify differentially expressed genes (DEGs) at two time points (seeds 30 days after the blooming period and 90 days after the blooming period). Gene functional enrichment analyses of DEGs identified two fatty acid synthesis-related genes and seven nervonic acid synthesis-related genes were identified by GO and KEGG analysis. The results elucidated the mechanism of fatty acid synthesis in *A. truncatum* seeds and can be used to facilitate *A. truncatum* breeding and intensive cultivation.

## Results

### Transcriptome sequencing of *A. truncatum* seeds containing contrast fatty acid

To identify genes expressed in *A. truncatum* seeds with different fatty acid, we constructed 12 cDNA libraries from the low fatty acid strain Y38 and the high fatty acid strain YQC, with seeds at 30 days after blooming period (Y38_1 and YQC_1) and 90 days after blooming period (Y38_2 and YQC_2). The libraries were sequenced with an Illumina Novaseq 6000 sequencing platform (Illumina, USA). A total of 70.52 Gb clean data were obtained, with an average of 5.88 Gb per library. Approximately 88.99% of the clean reads in all libraries were mapped to the *A. truncatum* reference genome. The Q30 bases were greater than 90% for each sample, and the GC content ranged from 44 to 45% (Table S2). The principal component analysis and Pearson correlation coefficients between samples revealed that the same stage of seed development was grouped together (Figure S1), indicating high similarity of their transcriptome expression.

### Identification of differentially expressed genes in *Acer truncatum* seeds at different stages

**Fig. 1:**
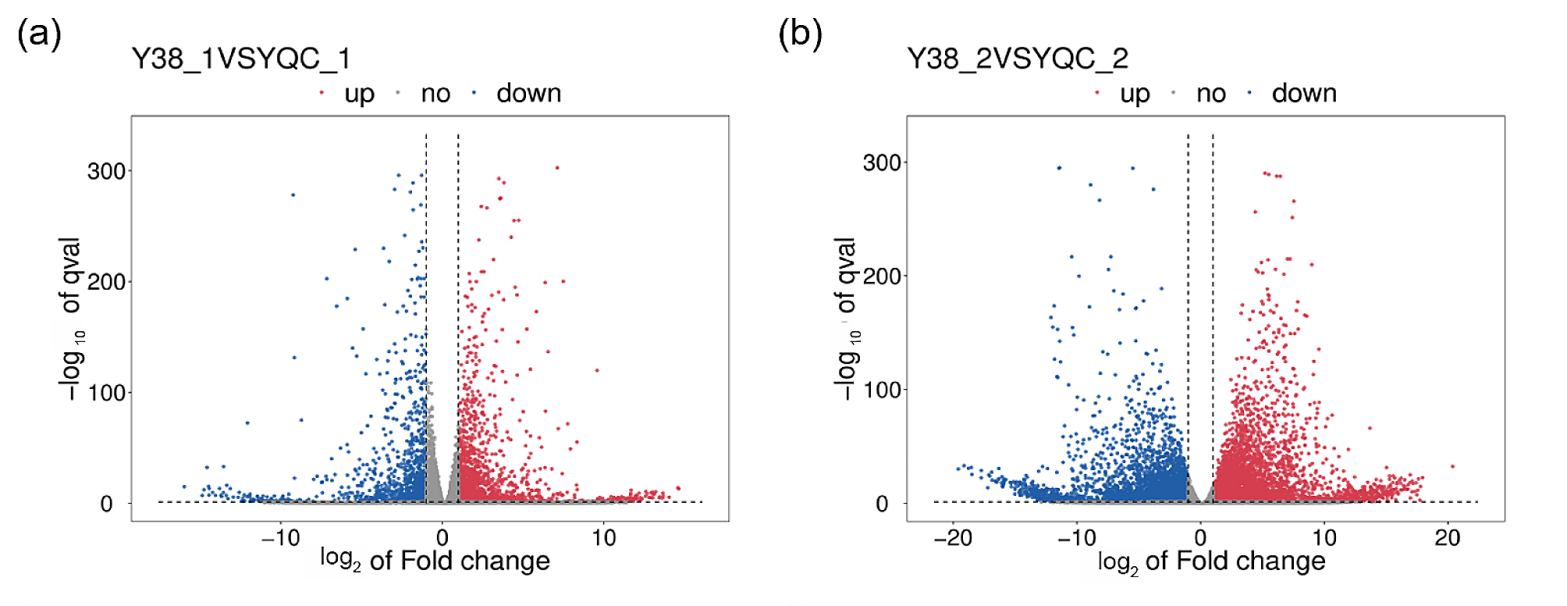
Volcano map of differentially expressed genes among sample groups of *Acer truncatum*

**Fig. 2:**
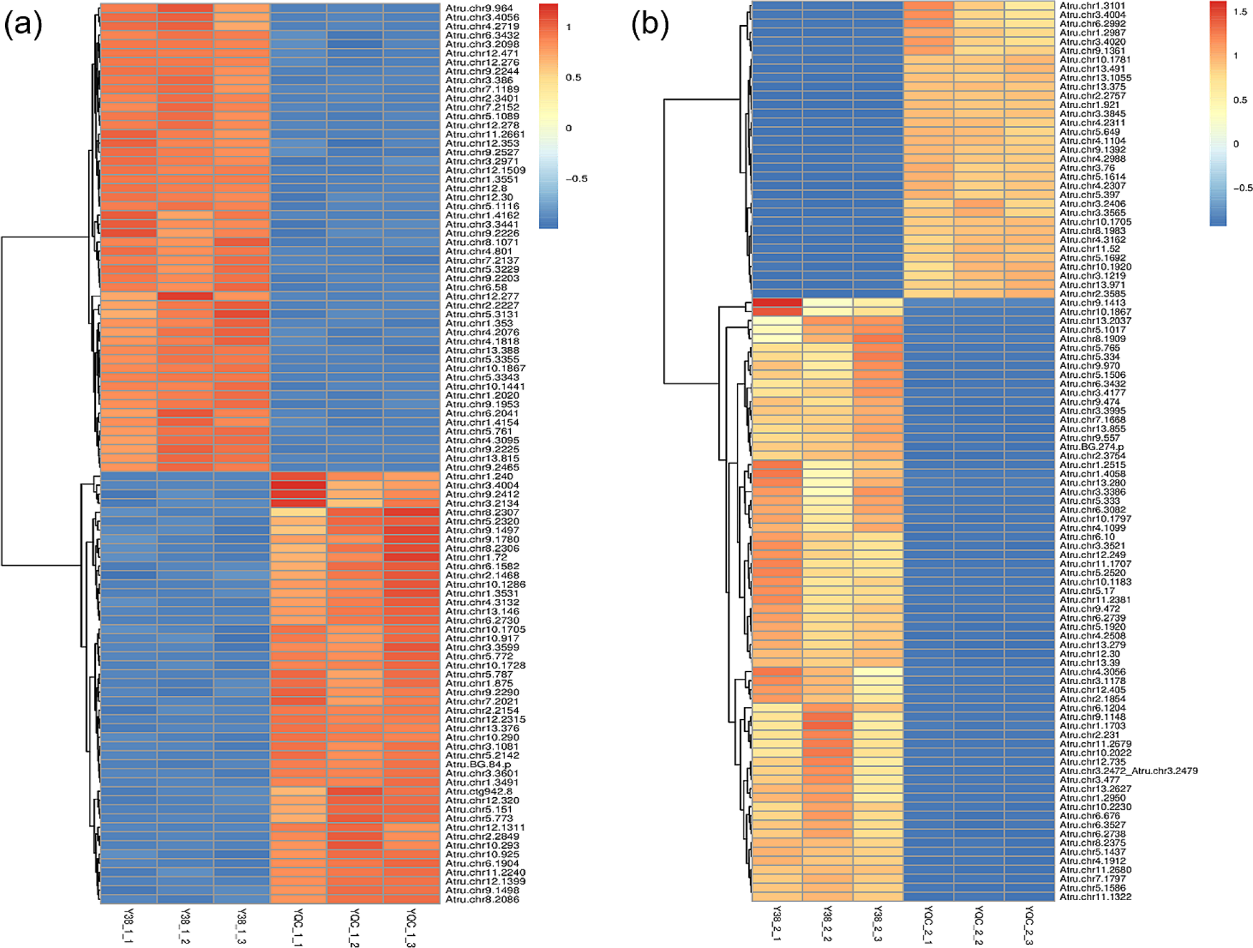
Heat map of differential gene clustering Note: The horizontal coordinate of the heat map is the sample, and the vertical coordinate is the screened differentially expressed genes (the default Top100 with the smallest q-value is used as an example of the heat map); different colors indicate different gene expression levels, from blue through white to red indicating low to high expression; red indicates highly expressed genes and blue indicates low expressed genes. It is important to note that the Z-value normalization can only be used to visually compare the expression levels of the same gene in different samples by color, not different genes

The genes that were differentially expressed between the Y38 seeds and the YQC seeds at different developmental stages were analyzed using the Hisat2. After mapping the clean reads of *A. truncatum* to the reference genome, we identified 3,618 DEGs in Y38_1 vs. YQC_1 seeds at 30 days after blooming period, including 2,333 up-regulated and 1,285 down-regulated genes; 9,340 DEGs between Y38_2 and YQC_2 seeds at 90 days after blooming period were identified, including 5,422 up-regulated and 3,918 down-regulated genes (Table S3, Figs. 1 and 2). The number of DEGs and upregulated DEGs of Y38 vs. YQC seeds in the later stages of development (90 days after the blooming period) was greater than that in the early stage (30 days after the blooming period).

### GO enrichment analysis of DEGs

**Fig. 3:**
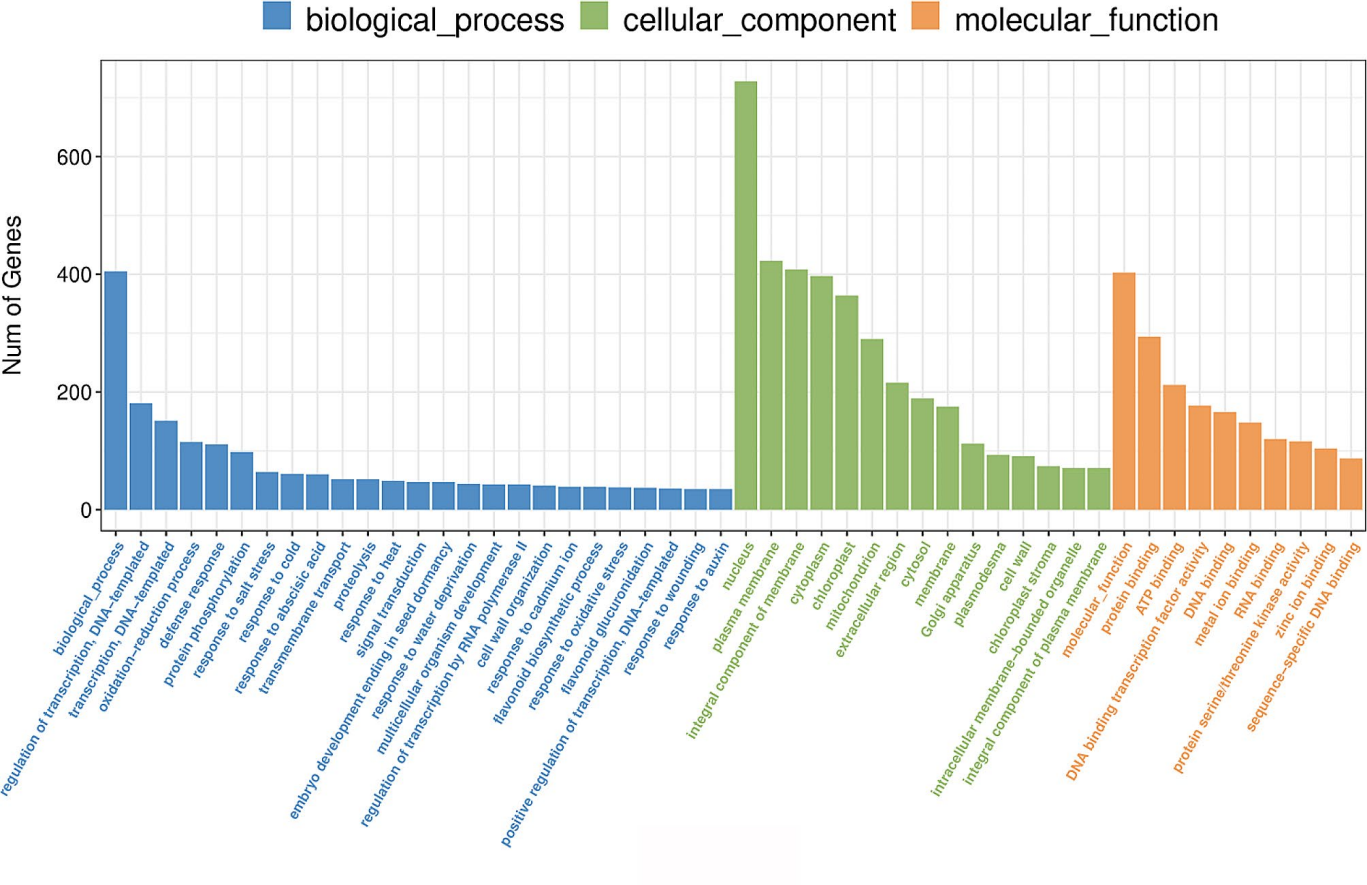
Gene Ontology (GO) classifcation of assembled unigenes in comparison group Y38_1 vs. YQC_1

**Fig. 4:**
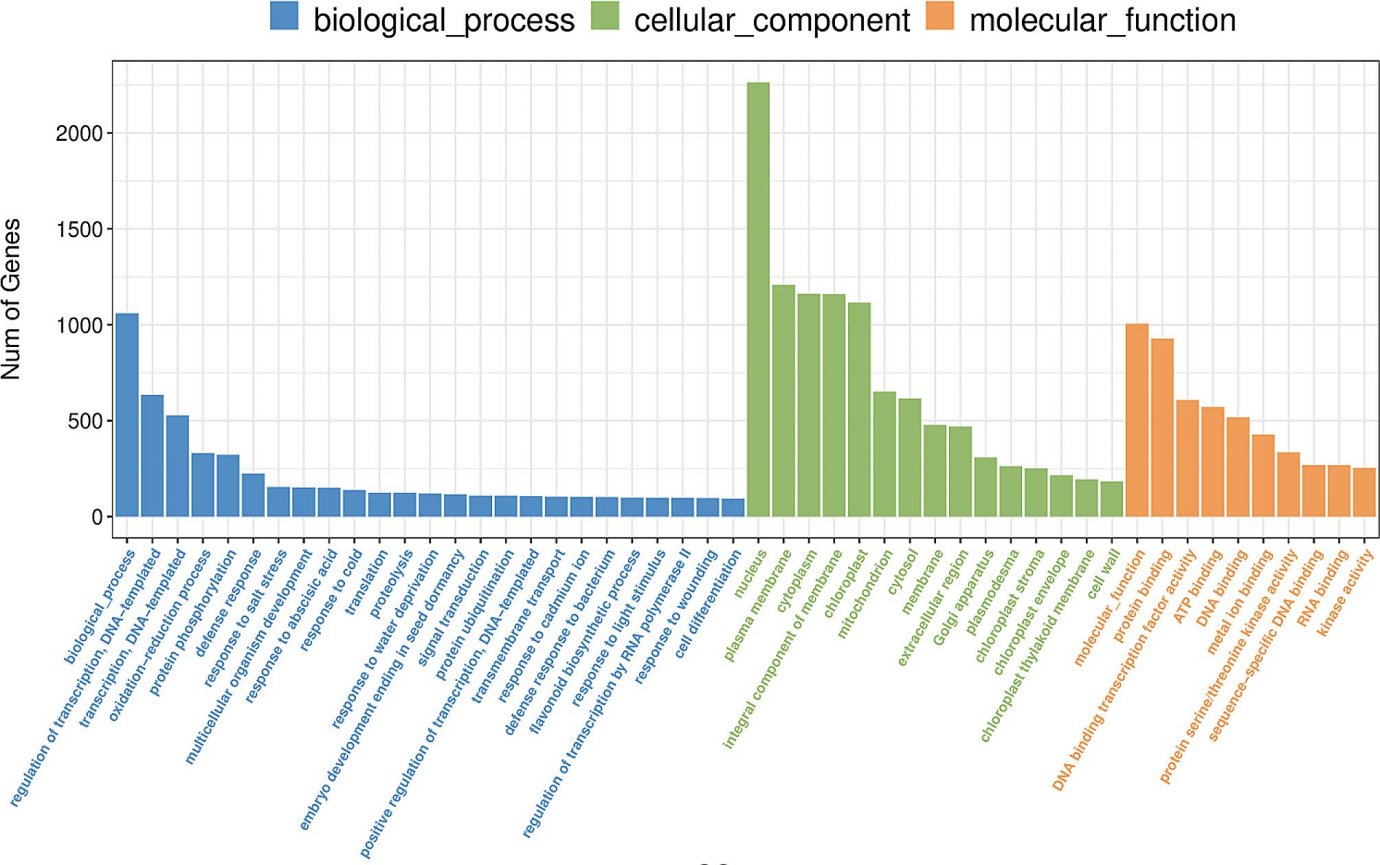
Gene Ontology (GO) classifcation of assembled unigenes in the comparison group Y38_2 vs. YQC_2

**Fig. 5:**
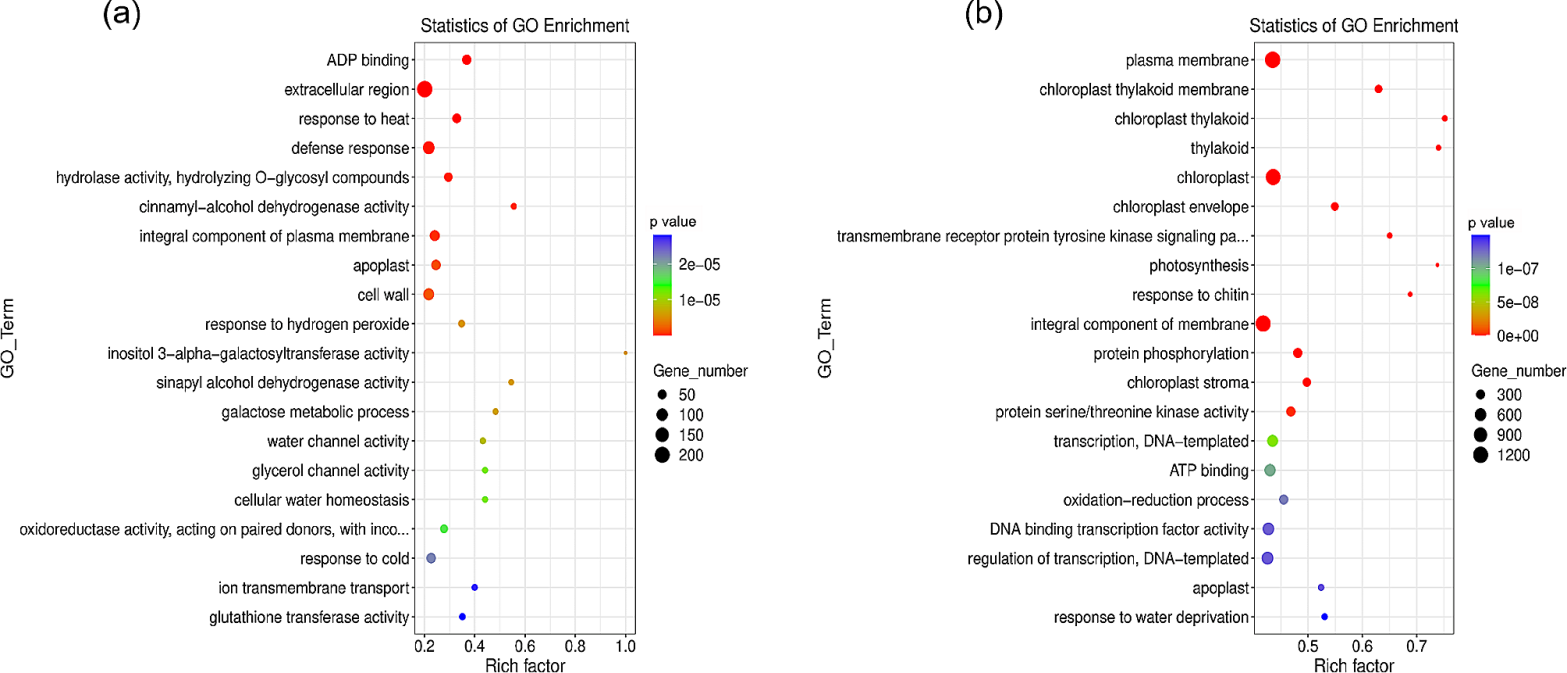
GO enrichment bubble diagram. **A** is the comparison group Y38_1 vs. YQC_1, **B** is the comparison group Y38_2 vs. YQC_2. Note: The horizontal coordinate Rich factor indicates the ratio of the number of differential genes located in the GO to the total number of genes located in the GO (Rich factor = S gene number / B gene number), the larger the Rich factor, the higher the degree of GO enrichment; the vertical coordinate is GO Term, i.e. GO functional annotation; in the bubble plot, the size of the bubble represents the S gene number, and the color of the bubble represents the p-value of the enrichment analysis, i.e. the significance of the enrichment, the smaller the p-value, the more significant the enrichment (same below)

To understand the function of DEGs in *A. truncatum* seeds, we performed a functional enrichment analysis for GO terms in the DEGs at two time points for Y38 vs. YQC seeds. The results showed that the DEGs at different developmental stages in Y38 vs. YQC seeds were enriched in many GO terms. We filtered the top 20 GO terms at each time point in which the DEGs were enriched (Figs. 3 and 4). The Y38_1 vs. YQC_1 group enriched genes mainly related to the GO terms like extracellular region, defense response, cell wall, and integral component of plasma membrane (Fig. 5a). In contrast, the Y38_2 vs. YQC_2 group enriched genes mainly involved in plasma membrane, chloroplast, integral component of membrane, and protein phosphorylation (Fig. 5b).

### KEGG enrichment analysis of DEGs

**Fig. 6:**
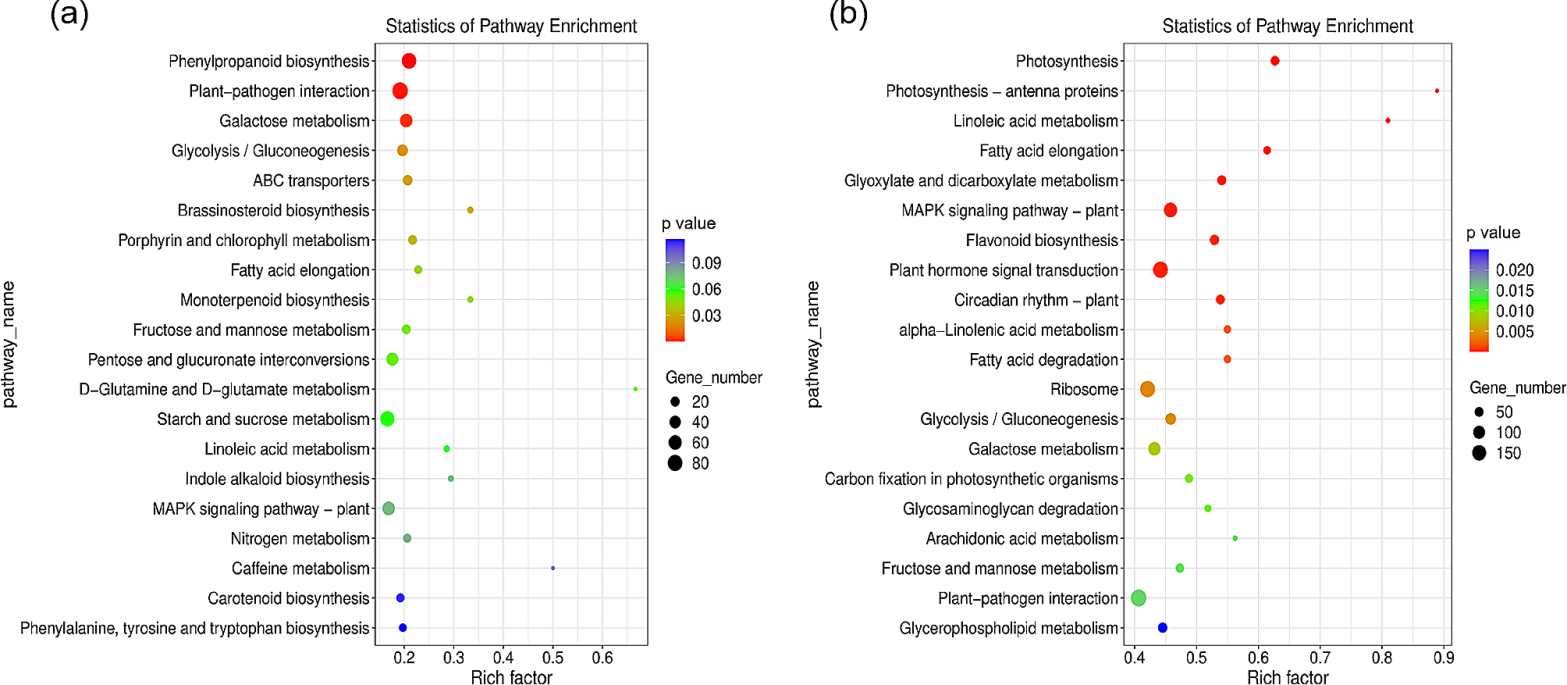
KEGG enrichment bubble diagram. **A** is the comparison group Y38_1 vs. YQC_1., **B** is the comparison group Y38_2 vs. YQC_2

To investigate metabolic pathways altered at different developmental stages of *A. truncatum* seeds, we performed KEGG pathway enrichment analyses in the DEGs at two time points for Y38 vs. YQC seeds. The top 20 significantly enriched pathways for DEGs in Y38_1 vs. YQC_1 and Y38_2 vs. YQC_2 were mentioned in Fig. 4a and b, respectively. The Y38_1 vs. YQC_1 group enriched genes mainly involved in Phenylpropanoid biosynthesis, Plant-pathogen interaction, and Galactose metabolism (Fig. 6a), while the Y38_2 vs. YQC_2 group mainly enriched genes mainly related to the Plant hormone signal transduction, MAPK signaling pathway, Flavonoid biosynthesis, Glyoxylate and dicarboxylate metabolism, and Photosynthesis. Interestingly, several pathways related to fatty acid metabolism, such as Linoleic acid metabolism, Fatty acid elongation, alpha-Linolenic acid metabolism, and Fatty acid degradation were also enriched in the Y38_2 vs. YQC_2 (Fig. 6b). These results indicated that the synthesis of fatty acids in *A. truncatum* seeds may occur in the later stages of development, and may be related to plant hormone transduction and photosynthesis.

### Analysis of fatty acid and nervonic acid-related gene expression levels

**Fig. 7:**
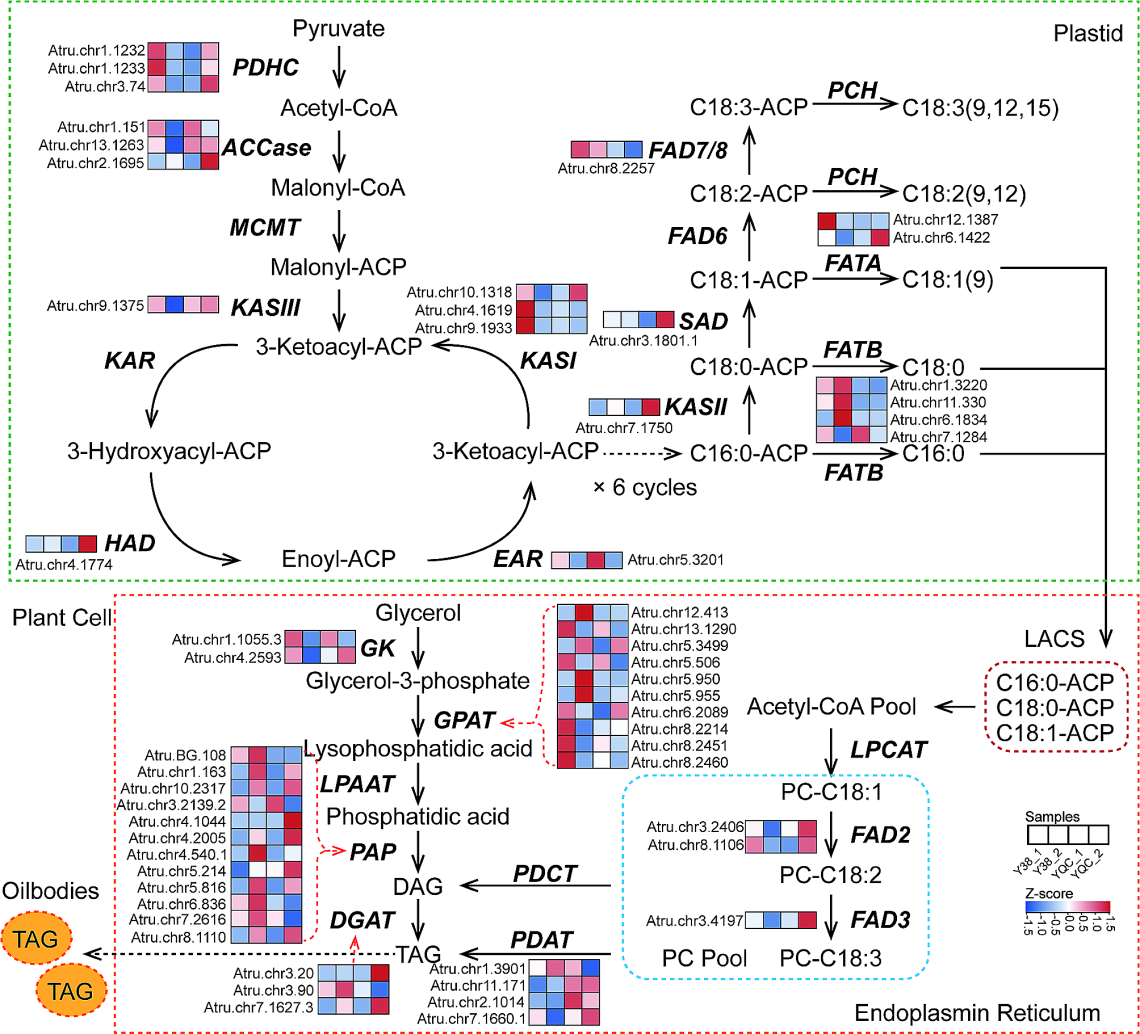
The metabolic pathway diagram of fatty acid synthesis

**Fig. 8:**
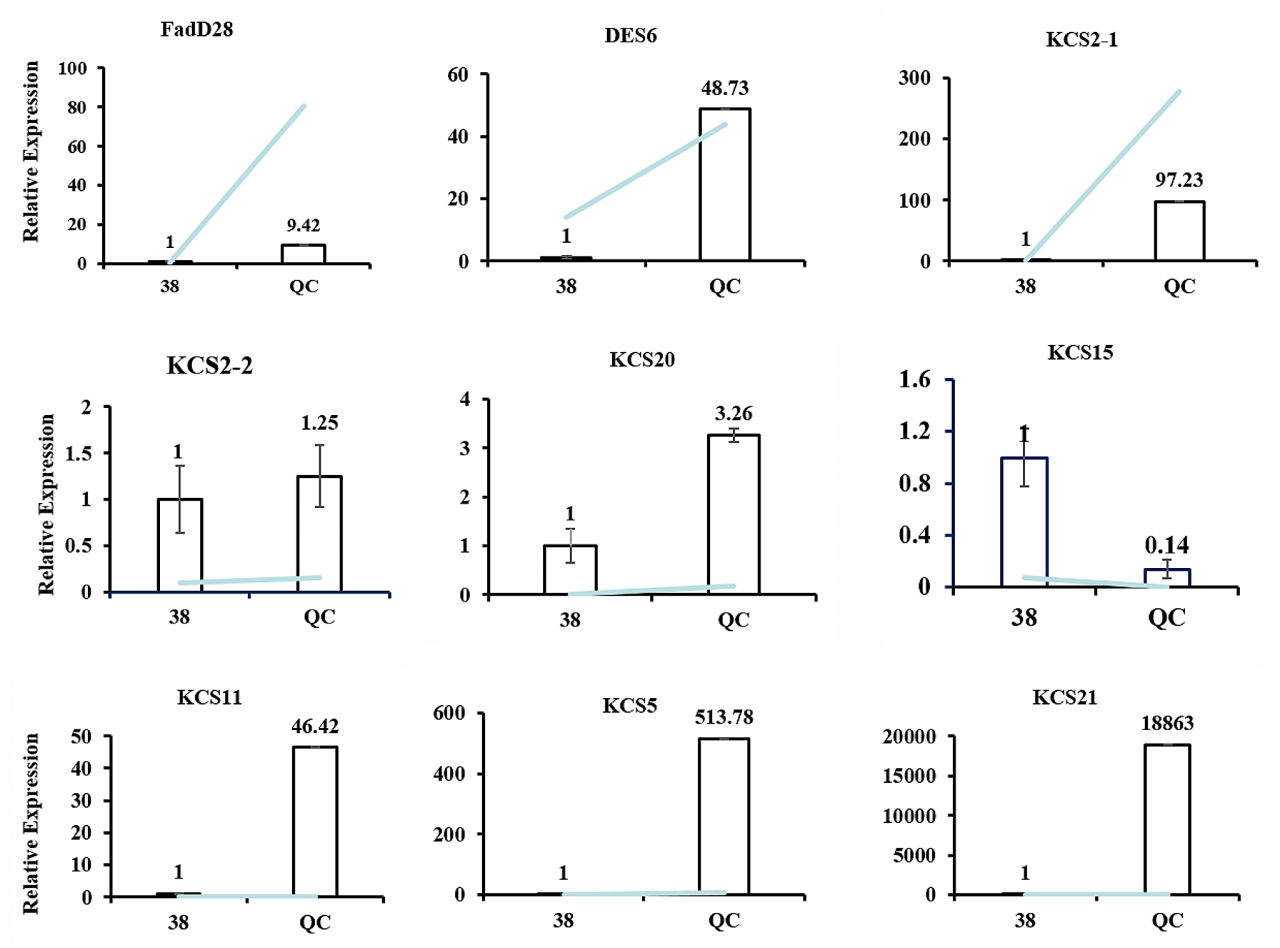
Expression of 9 fatty acid-related genes in the seeds of *A. truncatum*. Expression data were normalized against the data for the actin housekeeping gene and are presented as the mean ± standard error

To explore candidate genes related to fatty acid and nervonic acid synthesis during the development of *A. truncatum* seeds, a targeted analysis of the metabolic pathway diagram of fatty acid was conducted (Fig. 7). We found that FadD28 (*Atru.chr3.2513*, Long-chain-fatty-acid–AMP ligase FadD28) and DES6 (*Atru.chr13.1709*, Stearoyl-[acyl-carrier-protein] 9-desaturase 6) related to fatty acid synthesis and seven KCS (3-ketoacyl-CoA synthase) genes (*Atru.chr4.2308*, *Atru.chr4.2307*, *Atru.chr4.2304*, *Atru.chr7.1033*, *Atru.chr4.2306*, *Atru.chr11.2254*, and *Atru.chr4.2882*) related to nervonic acid significantly changed in gene expression according to the results of KEGG analysis of transcription group (Tables 1 and 2). Meanwhile, the above nine fatty acid-related genes were selected for qRT-PCR analyses to verify the reliability of transcriptome sequencing data. The qRT-PCR analyses showed that the expression of *FadD28*, *DES6*, *KCS2-1*, *KCS2-2*, *KCS20*, *KCS11*, *KCS21*, and *KCS5* in YQC were up-regulated compared to Y38 (Fig. 8). These qRT-PCR results were consistent with those of the transcriptome, indicating the reliability of the transcriptome data.

**Table 1:** Fatty acid synthesis genes

Gene id	Gene description	Y38-1VSYQC-1	Y38-2VSYQC-2
Atru.chr3.2513	Long chain fatty acid AMP ligase FadD28	1.55	-4.95
Atru.chr13.1709	Stearoyl-9-desaturase 6(DES6)	0.38	-6.12

**Table 2:** Neuroacid-related genes

gene_id	Gene description	Y38-1VSYQC-1	Y38-2VSYQC-2
Atru.chr4.2308	3-ketoacyl-CoA synthase 2 (KCS2)	-0.39	-12.38
Atru.chr4.2307	KCS2 KCS20 KCS15 KCS11 KCS5 KCS21	0.84	-11.84
Atru.chr4.2304		-2.46	-13.10
Atru.chr7.1033		-1.42	8.91
Atru.chr4.2306		1.14	-11.01
Atru.chr11.2254		1.81	-9.97
Atru.chr4.2882		0	9.62

## Discussion

*Acer truncatum* is an important oil-bearing woody tree and its kernel is rich in oil (42%), of which 85–93% is unsaturated fatty acids, which happens to be a source of nervonic acid (C24:1^Δ15^) (5%) [3].Evaluating the differences in oil content and fatty acid composition of germplasm resources is crucial for sustainable cultivating stable oil crops [17]. The oil content and fatty acid composition of *A. truncatum* populations from different regions in China have been widely reported. Qiao et al., analyzed the seed oil of 138 materials from 14 populations of *Acer truncatum* (Aceraceae family) native to China and found that the oil content ranged from 17.81 to 36.56% (mean: 28.57%), which mainly consisted of 14 types of fatty acids, and the nervonic acid content ranged from 3.90 to 7.85% among the accessions [18]. We previously analyzed the oil content and fatty acid composition of 22 *Acer truncatum* germplasm resources in Shandong Province, and found that the oil content and nervonic acid contents of cultivar ‘QC’ was 38.9% and 9.68%, respectively, while ‘38’ exhibited low fatty acid (oil content is 25.7%, nervonic acid contents is 4.79%)in the seed oil [19]. Therefore, the high fatty acid strain YQC and low fatty acid strain Y38 were used as the test materials to performed a comparative transcriptome analysis.

The continuous development of sequencing technology and bioinformatics has provided convenience for exploring genes and pathways related to fatty acid synthesis in oil crops [20–22]. In sesame (*Sesamum indicum* L.) seeds, the regulatory co-expression network was conducted and the FAD2, LOC10515945, LOC105161564, and LOC105162196 genes were identified to regulate the accumulation of unsaturated fatty acid biosynthesis [14]. In oil palm (*Elaeis guineensis* Jacq.), the expression profiles of genes in the fatty acid (FA) and triacylglycerol (TAG) biosynthesis processes in interspecific hybrids and identified the genes encoding key enzymes involved in the FA and TAG synthesis pathways through the transcriptome data [15]. In this study, we identified 3,618 DEGs in Y38_1 vs. YQC_1 seeds and 9,340 DEGs between Y38_2 and YQC_2 seeds. Gene functional enrichment analyses of DEGs found that several pathways related to fatty acid metabolism, such as Linoleic acid metabolism, Fatty acid elongation, alpha-Linolenic acid metabolism, and Fatty acid degradation were also enriched in the Y38_2 vs. YQC_2. These results indicated that the synthesis of fatty acids in *A. truncatum* seeds may occur in the later stages of development, and may be related to plant hormone transduction and photosynthesis. Meanwhile, FadD28 and DES6 related to fatty acid synthesis were identified, which provided gene reserves for subsequent functional gene validation.

Nervonic acid is a very long chain of monounsaturated omega-9 fatty acid chiefly found in nervous and brain tissues, which was reported to be related to psychiatric disorders [5–7]. The nervonic acid was only contained in a few known plants such as *Malania oleifera*, *Lunaria annua*, *Borago officinalis*,*Cannabis sativa*, *Tropaeolum speciosum*, *Cardamine graeca*, and *Xanthoceras sorbifolium* [23, 24]. As the first rate limiting enzyme involved in the fatty acid carbon chain elongation reaction, 3-ketoacyl-CoA synthase (KCS) plays an important role in the biosynthesis of nervonic acid. Seed-specific expression of the MoKCS11 from *M. oleifera* in Arabidopsis thaliana led to about 5% nervonic acid accumulation [25]. Compared to 2.8% in wild type plant, the highest nervonic acid level in transgenic *B. carinata* expressing the *Lunaria* KCS reached 30% [26]. In this study, we identified seven *KCS* genes related to nervonic acid. Among them, the expression of *KCS2-1*, *KCS2-2*, *KCS20*, *KCS11*, *KCS21*, and *KCS5* in YQC were up-regulated compared to Y38 by qRT-PCR, which was possible to conduct in-depth research as a candidate gene for promoting nervonic acid synthesis in the future.

## Conclusions

In this study we generated the comparative transcriptome analysis in high fatty acid strain YQC and those from low fatty acid strain Y38 at two developmental stages. Compared with YQC_1, a total of 3,618 DEGs were identified, including 2,333 up-regulated and 1,285 downregulated DEGs in Y38_1. In the Y38_2 vs. YQC_2 comparison group, 9,340 genes were differentially expressed, including 5,422 up-regulated and 3,918 down-regulated genes. Gene functional enrichment analyses of DEGs found that the synthesis of fatty acids in *A. truncatum* seeds may occur in the later stages of development, and may be related to plant hormone transduction and photosynthesis. FadD28 and DES6 related to fatty acid synthesis were identified. Meanwhile, *KCS2-1*, *KCS2-2*, *KCS20*, *KCS11*, *KCS21*, and *KCS5* in YQC may promote the accumulation of nervonic acid. These results provide a theoretical basis and gene reserve for the genetic improvement of *Acer truncatum*.

## Methods

### Plant material

The seeds of *A. truncatum* cultivars ‘QC’ and ‘38’ were collected from the experimental nursery of Shandong Provincial Academy of Forestry. The *A. truncatum* cultivar ‘QC’ exhibited a high fatty acid (oil content is 38.9%, nervonic acid contents is 9.68%), and ‘38’ exhibited low fatty acid (oil content is 25.7%, nervonic acid contents is 4.79%) in the seed oil based on years of content determination [19]. There were four kinds of samples: Y38_1, Y38_2, YQC_1, and YQC_2, divided into two comparison groups (Y38_1 vs. YQC_1 and Y38_2 vs. YQC_2) to compare the differences between the groups. Each group contained three independent replicates.

### RNA sequencing

Total RNA was isolated from each sample by TRIzol reagent (Thermo Fisher Scientific, USA) according to the manufacturer’s instructions. The purity and integrity of total RNA were evaluated by nanodrop ND-1000 (Nanodrop, USA) and Bioanalyzer 2100 (Agilent, USA). The total RNA meeting concentrations > 100 ng/µL and RNA integrity number (RIN) > 7.0 was used for following sequencing library construction. The RNA sequencing library with an average insert size of 300 bp was constructed by the TruSeq RNA Library Prep Kit v2 (Illumia, USA) following the manufacturer’s instructions. Then, the constructed libraries were sequenced on an Illumina Novaseq 6000 sequencing platform (Illumina, USA) with a paired-end (PE) 150 bp sequencing mode following the vendor’s recommended protocol.

### RNA sequencing data analysis

The raw RNA sequencing data was first trimmed and filtered by Trimmomatic version 0.39 [27] with default parameters. The generated clean data was then mapped to the *A. truncatum* reference genome version 2 (10.6084/m9.figshare.12986237.v2) [21] by HISAT2 version 2.2.1 [28] with default parameters. Gene abundance was quantified by eatureCounts version 2.0.3 [29]. The principal component analysis and Pearson correlation coefficients on gene abundance were performed by vegan version 2.6–4 package in R version 4.2.

### Identification of differentially expressed genes

The identification of differentially expressed genes (DEGs) was performed by DEseq2 version 1.34.0 [30], and the genes with fold change > 2 and P value < 0.05 were considered as DEGs. Gene Ontology (GO) and Kyoto Encyclopedia of Genes and Genomes (KEGG) pathway enrichment analyses of DEGs were conducted by clusterProfiler version 3.10.1 [31] using all genes as background. Gene functional enrichment data was visualized by ggplot2 (https://ggplot2.tidyverse.org.) in R version 4.2.

### Quantitative real-time reverse-transcription PCR

The fatty acid-related DEGs selected were validated by quantitative real-time reverse-transcription polymerase chain reaction (qRT-PCR). The primers (Table S1) for DEGs were designed by Primer Premier 5 and synthesized by Shanghai Shenggong Bioengineering Co., Ltd. The cDNA synthesis was performed using a reverse transcription kit (Vazyme, Nanjing, China). The qRT-PCR reactions were performed on a Bio-Rad CFX Connect Real-Time instrument using SYBRGreen fluorescent dye (Vazyme, Nanjing, China) according to the vendor’s recommended protocol.

### Electronic supplementary material

Below is the link to the electronic supplementary material.

Supplementary Material 1

Supplementary Material 2

## Data Availability

The data sets are included within the article and its Additional files. The RNA sequencing data are deposited in the NCBI under accession PRJNA1018686.
