# Supplementary material for: Transcriptome analyses of Acer Truncatum Bunge seeds to delineate the genes involved in fatty acid metabolism
Source: BMC Genomics. 2024 Jun 17;25:605. doi: 10.1186/s12864-024-10481-1 (PMC11181630; doi:10.1186/s12864-024-10481-1)
Supplement: Supplementary file 1 — Supplementary Material 1 [file 12864_2024_10481_MOESM1_ESM.docx]

**Table S1 Quantitative primer names and sequences**

| Primer name | Sequence |
| --- | --- |
| Atru.chr3.2513F | CCTTATGGTTGCTGCTGAAC |
| Atru.chr3.2513R | CCCTCCTCCATATGCACAGC |
| Atru.chr13.1709F | CACCATGCAAGCCTCACA |
| Atru.chr13.1709R | CTGGGGTTTGAGTAGTGGCG |
| Atru.chr4.2308F | CTCGTACCACTCCTTGTCG |
| Atru.chr4.2308R | CGTGAACGTCCCTGCTCTT |
| Atru.chr4.2307F | GAAGGTCAATGTCGGATGC |
| Atru.chr4.2307R | GTCGGATTGAACAGGCTGC |
| Atru.chr4.2304F | CTCGGTTGTGCACTAAAG |
| Atru.chr4.2304R | CCCGTAAGTCATTTGCCC |
| Atru.chr7.1033F | CTCACCTAGCTTCTCCGTCC |
| Atru.chr7.1033R | GTAGATAGGTAGAGCGAGGTG |
| Atru.chr4.2306F | GCTCCTACCAGCCGTTCTT |
| Atru.chr4.2306R | TGTGAACGTCCCTGCTCC |
| Atru.chr11.2254F | GCCCTTCACCTTCTCTCTCC |
| Atru.chr11.2254R | GGACAACAAGATCGCTGCACTG |
| Atru.chr4.2882F | CGCCTACACCGTCTCTAAG |
| Atru.chr4.2882R | GACAATAAGATGGCTGCTC |
| GAPDH-F | TCAACAATGCCAAACCTG |
| GAPDH-R | GTGTCAACGAGCACGAAT |

**Table S2 Data statistics of transcriptome Illumina sequencing data of Acer truncatum samples**

| Sample | Raw Data | | Valid Data | | Valid Ratio  (reads) | Q20% | Q30% | GC content% |
| --- | --- | --- | --- | --- | --- | --- | --- | --- |
|  | Read | Base | Read | Base |  |  |  |  |
| Y38_1_1 | 40925474 | 6.14G | 34127548 | 5.12G | 83.39 | 99.97 | 97.99 | 44 |
| Y38_1_2 | 49523674 | 7.43G | 41453030 | 6.22G | 83.70 | 99.96 | 98.00 | 44 |
| Y38_1_3 | 47866022 | 7.18G | 39761946 | 5.96G | 83.07 | 99.97 | 97.97 | 44 |
| Y38_2_1 | 43312032 | 6.50G | 39844020 | 5.98G | 91.99 | 99.96 | 97.70 | 44 |
| Y38_2_2 | 42760484 | 6.41G | 37407574 | 5.61G | 87.48 | 99.96 | 97.71 | 44 |
| Y38_2_3 | 43282324 | 6.49G | 39092834 | 5.86G | 90.32 | 99.96 | 97.78 | 44 |
| YQC_1_1 | 41510780 | 6.23G | 36839992 | 5.53G | 88.75 | 99.95 | 97.79 | 44 |
| YQC_1_2 | 40697576 | 6.10G | 36495994 | 5.47G | 89.68 | 99.97 | 97.90 | 44 |
| YQC_1_3 | 43815834 | 6.57G | 38113060 | 5.72G | 86.98 | 99.98 | 98.15 | 44 |
| YQC_2_1 | 43003136 | 6.45G | 37995798 | 5.70G | 88.36 | 99.96 | 97.78 | 44.50 |
| YQC_2_2 | 44868862 | 6.73G | 43586818 | 6.54G | 97.14 | 99.95 | 97.15 | 45 |
| YQC_2_3 | 46845826 | 7.03G | 45422078 | 6.81G | 96.96 | 99.93 | 97.04 | 44.50 |

**Table S3 Analysis of differentially expressed genes among sample groups of Acer truncatum**

| Comparison between groups | Up-regulated genes | Down-regulated genes | Number of differential genes |
| --- | --- | --- | --- |
| Y38_1 VS YQC_1 | 2333 | 1285 | 3618 |
| Y38_2 VS YQC_2 | 5422 | 3918 | 9340 |
