# Supplementary figures and images for: Transcriptome analyses of Acer Truncatum Bunge seeds to delineate the genes involved in fatty acid metabolism
Source: BMC Genomics. 2024 Jun 17;25:605. doi: 10.1186/s12864-024-10481-1 (PMC11181630; doi:10.1186/s12864-024-10481-1)

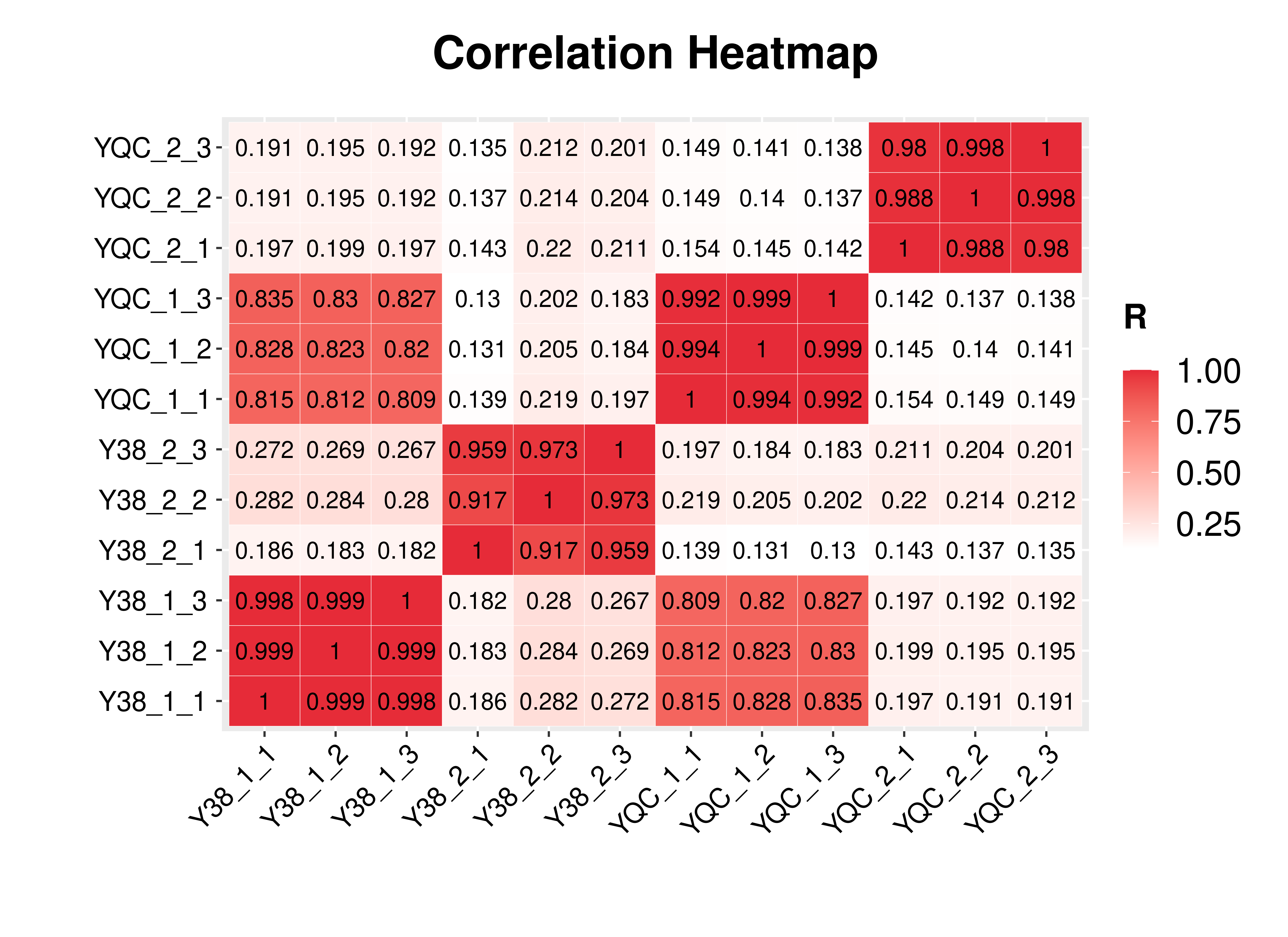

Supplement: Supplementary file 2 — Supplementary Material 2 [file 12864_2024_10481_MOESM2_ESM.png]
